# The relationship between Plasma Markers and Essential Hypertension in Middle-aged and Elderly Chinese Population: A Community Based Cross-sectional Study

**DOI:** 10.1038/s41598-019-43278-4

**Published:** 2019-05-02

**Authors:** Tesfaldet Habtemariam Hidru, Xiaolei Yang, Yunlong Xia, Li Ma, Hui-Hua Li

**Affiliations:** 1 School of Public Health, Dalian Medical University, Dalian, 116044 China; 2 Department of Cardiology, Institute of Cardiovascular Diseases, First Affiliated Hospital of Dalian Medical University, Dalian, 116011 China

**Keywords:** Prognostic markers, Risk factors

## Abstract

Plasma markers have been continuously advocated as pointers to estimate the long-term risk of cardiovascular disease in the general population. We examined the relationship between plasma high-sensitivity C-reactive protein (hs-CRP), homocysteine (Hcy), high-sensitivity cardiac troponin T (hs-cTnT), N-terminal prohormone of brain natriuretic peptide (NT-proBNP), 25-Hydroxyvitamin D (25OHD), glycosylated hemoglobin A1c (HbA1c), and serum uric acid (SUA) levels and hypertension in middle and old aged population. A total of 2624 Chinese (62.02 ± 5.73 years old) were recruited into a population-based, cross-sectional study. Plasma hs-CRP, Hcy, HbA1c, and SUA levels were significantly higher in the hypertension group compared with control in the entire population and men (P = 0.05 for all). We observed a positive association between the highest quartiles of Hcy, NT-proBNP, HBA1c concentrations, and the prevalence of hypertension, OR (95% CI) = 1.48 (1.16–1.90), 1.62 (1.27–2.07) and 1.94 (1.49–2.52), respectively. The multivariable-adjusted OR of hypertension for the fourth versus the first quartile of homocysteine were 2.00 and 1.39 in men and women, respectively. In conclusion, our study found an independent and robust association between elevated Hcy, NT-ProBNP, and HBA1c levels and prevalence of hypertension in the middle-aged and elderly Chinese population. A follow-up study is necessary to endorse the observed association.

## Introduction

Hypertension carries significant weight in a global health perspective, and it is highly prevalent in mainland China^1^. Despite significant advancement in the management of hypertension, fundamental pathogenic mechanisms are not yet modified by currently existing treatment modalities. In addition to the continuing surge in the prevalence of hypertension attributed to failure to implement effective counteractions to reduce hypertension associated burden, recent studies persisted to inform the positive relationship between high blood pressure and cardiovascular events (CVE) and mortality^2^. Thus, for the prevention and management of elevated blood pressure (BP), reliable information about the biomarkers of hypertension is essential.

Earlier studies have recognized the role of inflammatory pathways activation as an important pathological event in the development of hypertension^3^. This growing evidence suggests that systemic inflammation endorses endothelial damage, which further contributes to physiological alterations in the endothelium^4^. Moreover, the pro-inflammatory pathways are well-known for their tremendous influence at lipid metabolism, suggesting that there is an interaction between inflammation and dyslipidemia^5^. Inversely, lipids have been reported to influence an inflammatory reaction, and an unhealthy lifestyle with increased cholesterol consumption can aggravate the inflammatory reaction, eventually leading to the expansion of metabolic syndrome (MetS)^6^. MetS is an independent risk factor in the pathogenesis of several CVDs, including hypertension^7^. C-reactive protein (CRP), a plasma marker that indicates inflammation, has been proposed to link with an elevated risk of hypertension. An increase in MetS associated risk factors, such as excess cholesterol and obesity, predisposes to insulin resistance and increases the risk of CVE and mortality^1^. Epidemiologic studies reported an inverse association between circulating levels of 25-hydroxyvitamin D (25-OHD)- a biomarker of vitamin D status, and metabolic syndrome^8,9^. Another published article highlighted that low vitamin D level depletes the intracellular concentrations of calcium, and interferes with the level of insulin secretion by beta cells which consequently impairs glucose tolerance^10^. With insulin resistance, there is an increased level of inflammation, oxidative stress, vascular adhesion molecules expression, and diminished levels of nitric oxide, which result in persistent hypertension due to an increase in vascular hardening^11^. Interestingly, a recent meta-analysis has provided firm evidence about the relations between lower circulating 25-OHD levels and hypertension^12^. However, there has been a recent debate about the role of low 25-OHD in the development of hypertension. Besides, Hemoglobin A1c (HbA1c)-a marker recommended to determine long-term blood glucose levels, has been reported as a clue in predicting CVD risks including peripheral artery disease, stroke and heart attack among individuals with diabetes mellitus. A shred of existing evidence reported that masked hypertension is associated with an increased risk of CVD in diabetic patients, and the presence of diabetes is an independent risk factor for central blood pressure increase^13^.

The contribution of neurohormones in the progression of hypertension has led to new treatment modalities such as β-blockers and angiotensin-converting enzyme (ACE) inhibitors. N-terminal prohormone of brain natriuretic peptide (NT-proBNP), a cleavage product of brain natriuretic peptide (BNP), is the gold standard for the diagnosis of heart failure and also it is an established marker for the prediction of cardiovascular events^14^. Recently, a study has demonstrated that NT-proBNP mirrors the detrimental effect of high blood pressure on subclinical organ damage denoted by aortic stiffness, left ventricular hypertrophy (LVH), or renal damage^15^. Like the NT-proBNP, high sensitivity cardiac troponin T (hs-TnT) has also been extensively studied in heart failure^16^. A previous study reported elevated levels of hs-cTnT in prehypertension stages, and an increase in hs-cTnT level was reported as an effective predictor of prehypertension^17^. To the extent of our knowledge so far, the mechanisms underlying increased levels of hs-cTnT in hypertensive subjects without ischemic heart disease are not fully elucidated. However, a previous study hypothesized that increased blood pressure may induce increased diastolic and systolic wall stress, damage/reshape coronary microvascular structures, or exert increased extravascolic compressive strength^18^. Surprisingly, studies conducted by Milwidsky *et al*. showed an independent association between hs-TnT and MetS^19^.

Homocysteine (Hcy), a non-proteinogenic α-amino acid, is a hemostatic marker of coagulation activation and endothelial dysfunction. Increased Hcy levels can be genetically inherited and/or acquired^20^. For instance, methionine metabolism from endogenous protein or diet degradation substantiates Hcy formation^21^, which is further cleared from the body through the kidneys. A previous study has demonstrated that the noxious effect of methionine residues causes alterations in the vascular endothelium^22^. Hcy increases endothelial cell damage, reduces vessels flexibility, and influences hemostasis^23^, indicating that increased Hcy adds to inflammation and vascular remodeling during the pathogenesis of hypertension. Evidence available suggests a possible connection between hyperhomocysteinemia and hypertension^24^. However, to validate whether Hcy should be considered as a biomarker or a risk factor, replication of consistent findings is required from various studies.

In general, biomarkers can grossly be utilized for risk stratification and for evaluation of therapeutic responses in CVDs. To the best of our knowledge, the pathophysiological mechanisms of hypertension require precise and accurate profiling of biomarkers. This is crucial in the effective management and monitoring of the disease process of hypertension. However, population-based studies that focus on the relationship between a number of plasma markers that include inflammatory, glycemic and coagulation markers and hypertension are limited, and the few studies on the association among the aforementioned plasma markers and hypertension have reported contradictory results^25–32^. These studies had several limitations; some enrolled a wide range of age or non-Chinese population, others were focused on either men or women. The current study assists to highlight a set of specific markers for hypertension in middle and old age population in northeast China. Thus, our findings add reliable information on plasma biomarkers for hypertension in middle and old age categories to the existing literature. Also, the present study is based on baseline data for a newly established prospective cohort study, and documentation of the baseline plasma marker levels could serve as reference values for the future. Considering the role of inflammation, arterial stiffness, oxidative stress and metabolic syndrome in the development of hypertension, and the involvement of the aforementioned biomarkers with either the redox of hypertension or risk factors of hypertension, we hypothesized that increased plasma Hcy, NT-proBNP, hs-TnT, HbA1c, SUA, and hs-CRP levels are associated with the prevalence of hypertension. Therefore, the purpose of this study was to investigate whether the most recent routine inflammatory (hs-CRP), cardiac (Hcy, NT-proBNP, and hs-TnT) and glycemic markers (HbA1c and SUA) are associated with BP readings and prevalence of hypertension in middle and old age population in North China.

## Methods

### Study design

We conducted a baseline population-based comparative cross-sectional study based on a prospective cohort study of the relative factors of heart failure among elderly people (PCSRFHFEP) data. The PCSRFHFEP study is a newly established prospective cohort study, currently registered in Dalian, China (ChiCTR-1900021163) to assess the risk factors for heart failure among the elderly population. In this study, we determined serum levels of hs-CRP, Hcy, HbA1c, hs-cTnT, NT-proBNP, SUA and 25-OHD in 2624 participants. We further performed a gender-specific investigation to examine the relationship between plasma markers and hypertension.

### Participants

**Figure 1:**
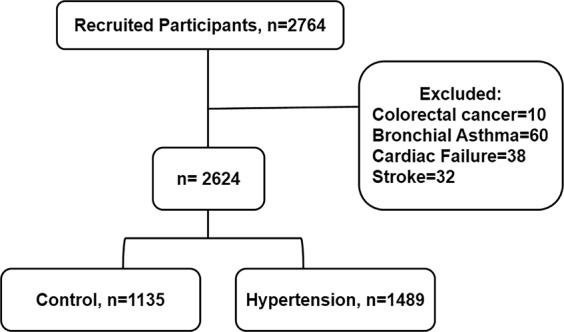
The flow chart of the recruitment and classification.

This study consists of 2764 Chinese participants enrolled between January 2017 and February 2018 in the PCSRFHFEP registries of Dalian City, Liaoning Province of China. We invited the retired workers of Dalian Port Authority to enroll for the PCSRFHFEP cohort study. Individuals above 50 years old with complete data, regardless of their gender status were eligible for the study. The eligible participants had a complete physical examination at Dalian Port Hospital. Exclusion criteria included: physician-diagnosed CVDs (history of stroke, congestive heart failure, coronary artery disease, myocardial infarction, and valvular heart disease), pacemaker implantation, severe cognitive or communication impairment, renal failure or insufficiency, liver cirrhosis, malignancy, active infectious/inflammatory disease and/or chronic obstructive pulmonary disease. One hundred and forty individuals did not fulfill the inclusion criteria, thus were excluded. Finally, 2624 individuals (1135 controls and 1489 cases of hypertension) were included in this study. This study was approved by First Affiliated Hospital of Dalian Medical University (approval number: LCKY2016-31), and informed consent was obtained prior to the data and specimen collection. The study protocol conformed to the law protecting personal data and agreed with the guidelines of Helsinki declaration. Cases of hypertension in the present study met at least one of the following conditions as described previously^25^: (1) systolic blood pressure (SBP) of >140 mmHg; (2) a diastolic blood pressure (DBP) of >90 mmHg; or (3) self-reported history of hypertension with the current use of blood pressure-reducing medication. Figure 1 demonstrates the flow chart of the recruitment and classification (Fig. 1).

### Baseline covariates

Basal data and various clinical details related to cardiovascular diseases were recorded following a detailed interview and physical examination. We gathered data on demography, health-related lifestyle, disease history, and use of any medication or drug. BP was measured twice using standard mercury sphygmomanometers with cuffs by trained physicians (measured from the brachial artery while the participant is seated in a chair for at least 5 min). If the first two records were quite different, additional measurements were made to minimize white coat hypertension. Diabetes mellitus was defined as current use of oral antidiabetic medications or insulin, fasting serum glucose >6.5 mmol/L after fasting for a minimum of 8 h, and/or a self-reported history of diabetes. Smoking exposure, alcohol consumption, and medication use were self-reported.

### Biological sampling and biochemical measurements

Blood samples were biochemically examined for the concentration of hs-CRP, Hcy, hs-cTnT, NT-proBNP, 25-OHD, and HBA1c. The investigators and laboratory personnel were not aware of the subjects’ health status, and all samples were treated identically throughout the processing and analysis. Plasma hs-CRP levels were measured by a nephelometric assay on a Behring Nephelometer II (BNII) analyzer (SIEMENS BNII). Plasma hs-TnT, NT-proBNP and 25-OHD concentrations were assayed using an electrochemiluminescence immunoassay (Roche Cobas e601). Serum HbA1c concentrations were determined by using high-performance liquid chromatography (Primer Hb9210^TM^-HPLC HBA1C analysis). Hcy levels were determined by using an enzymatic cycling method (Hitechi7600 series automatic analyzer). High-density lipoprotein cholesterol (HDL-C), triglycerides, low-density lipoprotein cholesterol (LDL), and serum total cholesterol were measured enzymatically.

### Data analyses

Study participants were compared by interquartile range or proportions of major hypertension risk factors, using student-t-test and Mann-Whitney-U test for normally and non-normally distributed data, respectively; and chi-square (*χ*^2^) test for categorical or ordinal data. The study population was stratified into quartiles based on plasma markers. Two-way analysis of variance (ANOVA) was used to detect if there was an interaction effect between genders and DM with hypertension status on the levels of plasma markers. Multiple linear regression analysis was employed to predict the systolic and diastolic BP levels (as continuous outcomes) based on the levels of markers’ concentration. Preliminary analyses were conducted to ensure no violation of assumptions of normality, homoscedasticity, independence, and linearity. Multicollinearity was assessed for all variables using tolerances and the variance inflation factors (VIF). Multicollinearity was detected when circulating biomarkers, BMI, lipid profiles and DM status were considered as an explanatory variable. The VIF of total cholesterol was greater than 10, indicating that there were high correlations among the independent (predictor) variables. However, neither assumptions were violated nor multicollinearity was detected following exclusion of total cholesterol from the model. As such, the multiple linear regression analysis was adjusted for age, gender, physical activity, cigarette status, BMI, alcohol use, triglycerides, HDL, LDL, creatinine, and DM. Binary logistic regression models were estimated, and the OR at 95% CIs were used to approximate the associated risk for hypertension according to quartiles of plasma markers, with the lowest quartile serving as the reference category. Model 1 was adjusted for age and gender. Model 2 was adjusted for age, gender, cigarette status, alcohol use, and physical activity. Model 3 was adjusted for the covariates in model 2, followed BMI, TC, HDL, LDL, TG, creatinine, and DM. Further, we ran a gender-specific analysis and sensitivity analysis to examine the relationship between plasma markers and hypertension. We performed all statistical analyses using SPSS software (IBM SPSS) version 21.

## Results

### Baseline characteristics

This study included 2624 individuals (1135 controls and 1489 cases) aged between 50 and 84 years. Baseline characteristics of participants are presented in Table 1. The values of the median SBP, DBP, creatinine, triglyceride, and LDL were significantly higher in the hypertension group compared with control (P < 0.01). Conversely, the median value of total cholesterol was not statistically different between hypertension and control group. The proportion of participants who were diagnosed with diabetes was significantly higher in the hypertension group compared with control. There was no statistically significant difference in physical activity between the two groups. Only 314(21.1%) of the hypertension cases were using antihypertensive drugs.

**Table 1:** Baseline characteristics of the participants, N = 2624.

Characteristic	Control (n = 1135)	Hypertension (n = 1489)	P
Male, N(%)	436(38.4%)	669(44.5%)	0.001
Age, years (Median)	62(58–65)	62(59–65)	0.016
BMI, mean ± SD, kg/m^2^	23.91 ± 3.02	25.48 ± 3.16	<0.001
Smoking status			
Never	823(72.5%)	975(65.5%)	<0.001
Former	100(8.8%)	232(15.6%)	
Current	212(18.7%)	282(18.9%)	
Alcohol use, N (%)			
Never	840(74%)	1001(67.2%)	0.001
Former	72(6.3%)	111(7.5%)	
Current	223(19.6%)	377(25.3%)	
SBP(mmHg)	128(120–134)	155(145–170)	<0.001
DBP(mmHg)	77(71–81)	90(83–97)	<0.001
Diabetes, N(%)	59(5.2%)	121(8%)	0.005
Fasting glucose (mg/dl)	5.53(5.16–6.05)	5.58(5.36–6.57)	<0.001
Physical activity			
Never	124(10.9%)	154(10.3%)	0.858
Sometimes	516(45.5%)	648(46.1%)	
Often	495(43.6%)	653(43.5%)	
Total Cholesterol, mean ± SD, mmol/L	5.39(4.72–6.02)	5.43(4.80–6.06)	0.201
TG, mean ± SD, mmol/L	1.16(0.84–1.62)	1.37(0.99–1.94)	<0.001
HDL-c, mean ± SD, mmol/L	1.45(1.22–1.71)	1.36(1.16–1.60)	<0.001
LDL-c, mean ± SD, mmol/L	3.3(2.76–3.87)	3.38(2.89–3.96)	0.008
Creatinine, mean ± SD, umol//l	62.62(57.10–73.50)	64.48(57.17–75.01)	<0.001
Antihypertensive use, N(%)^#^		314(21.1%)	
β-blockers	—	59(4%)	
ACEI	—	10(0.7%)	
ARB	—	63(4.2%)	
Calcium antagonists	—	136(9.1%)	
Diuretics	—	4(0.3%)	
Other Antihypertensive	—	70(4.2%)	

TG, Triglycerides; HDL-c, High-density lipoprotein; LDL-c, Low-density lipoprotein; ACEI, Angiotensin-converting enzyme inhibitors; ARB, Angiotensin receptor blockers.

^#^The figures and percentile for anti-hypertensive drugs describe the N(%) within hypertension group.

### Comparison of plasma markers and the interaction effect of gender and DM with hypertension status on the levels of the plasma markers

Plasma markers including, hs-CRP, Hcy, SUA, and HbA1c were statistically significantly higher in the hypertension group than in the control in the entire population, men and women (Table 2). Also, the concentrations of NT-proBNP were significantly higher in the hypertension group than in the control group in women. However, we found no statistically significant differences in 25-OHD levels between hypertension and control group in the entire population, men, and women.

**Table 2:** Comparison of the plasma markers between hypertension and control group.

	General population			Men			Women		
	Control (n = 1135)	Hypertension (n = 1489)	P	Control (n = 436)	Hypertension (n = 669)	P	Control (n = 699)	Hypertension (n = 820)	P
hs-CRP (mg/L)	0.86(0.47–1.71)	1.13(0.60–2.08)	<0.001	0.90(0.48–1.90)	1.11(0.61–2.16)	0.001	0.84(0.45–1.65)	1.15(0.59–2.03)	<0.001
Hcy (µmol/L)	9.11(7.80–11.36)	9.75(8.14–12.45)	<0.001	10.44(8.80–12.49)	11.24(9.14–14.17)	<0.001	8.54(7.45–10.17)	8.85(7.64–10.68)	0.004
NT-proBNP (ng/mL)	49.57(30.12–77.49)	51.32(31.37–87.57)	0.096	43.05(24.98–73.27)	44.59(26.53–79.39)	0.360	54.50(34.41–80.09)	57.89(36.55–91.93)	0.034
25-OHD (nmol/L)	13.74(9.92–18.63)	14.16(9.80–19.85)	0.168	16.23(11.65–23.01)	16.74(12.25–22.52)	0.356	12.46(8.89–16.64)	12.13(8.49–16.94)	0.733
HBA1C (%)	4.99(4.66–5.43)	5.19(4.77–5.84)	<0.001	5.03(4.64–5.44)	5.17(4.76–5.85)	<0.001	4.98(4.67–5.43)	5.22(4.80–5.82)	<0.001
hsTnT (ng/mL)	7.75(5.65–10.05)	8.04(6.01–10.36)	0.027	8.53(6.27–11.35)	9.17(6.73–11.84)	0.104	7.24(5.31–9.25)	7.26(5.55–9.50)	0.438
SUA (µmol/L)	297.85(253.47–352.47)	321.68(265.01–376.28)	<0.001	342.81(297.02–394.23)	357.34(308.57–409.68)	0.004	274.95(239.40–313.81)	287.06(246.15–339.70)	<0.001

Note: Plasma markers are expressed as Median (25% quartiles–75% quartiles). hs-CRP = high-sensitivity C-reactive protein; hs-cTnT, high-sensitivity cardiac troponin T; NT-proBNP, N-terminal prohormone of brain natriuretic peptide; 25OHD, 25-Hydroxyvitamin D; SUA, serum uric acid and HbA1c, glycosylated hemoglobin A1c.

A two-way ANOVA was considered to examine the effect of sex and DM with hypertension status on biomarker levels. There was a statistically significant interaction between the effects of DM and hypertension status on HBA1c levels, F (1, 2620) = 4.771, P = 0.025. Also, there were statistically significant differences in the NT-proBNP, hcy, hs-TnT, 25-OHD and SUA levels between male and female gender (p < 0.05). In contrast, no significant interaction between gender and hypertension status on plasma marker levels (P > 0.05) was observed. The effect of gender and DM with hypertension status on biomarker levels is presented in Table 3.

**Table 3:** The effect of gender, DM, and Hypertension status on biomarker levels.

Biomarkers	The effect of gender and Hypertension status			The effect of DM and Hypertension status		
	Type III Sum of Squares	F	P-value	Type III Sum of Squares	F	P-value
hs-CRP (mg/L)	5.142	0.181	0.670	2.900	0.102	0.749
HCY (µmol/L)	77.729	3.025	0.082	0.672	0.024	0.877
NT-proBNP (ng/mL)	16966.308	0.498	0.481	27752.051	0.813	0.367
25-OHD (nmol/L)	8.937	0.162	0.687	13.547	0.226	0.634
HBA1c (%)	0.695	0.530	0.467	4.771	5.004	0.025
SUA (µmol/L)	161.331	0.033	0.856	9940.076	1.663	0.197
hs-TnT (ng/mL)	70.356	0.690	0.406	0.003	0.000	0.996

Note: Degree of freedom (DF) for the effect of gender and hypertension status was 1 whereas DF for the effect of DM and Hypertension status was 2.

### The relationship between diastolic and systolic BP and plasma markers

In adjusted linear regression analysis, there was a positive association between DBP and Hcy (β = 0.073 µmol/L, 95% CI = 0.080–0.258; P < 0.001), 25-OHD (β = 0.039 nmol/L, 95% CI = 0.002–0.124; P = 0.044), and HBA1c (β = 0.046%, 95% CI = 0.032–0.963; P = 0.036) concentrations per 1 mmHg increase in DBP. Similarly, the findings of the present study demonstrated a positive association between SBP and Hcy (β = 0.076 µmol/L, 95% CI = 0.156–0.475; P < 0.001), HBA1c (β = 0.098%, 95% CI = 1.02–2.69; P < 0.001), hs-TnT (β = 0.055 ng/mL, 95% CI = 0.031–0.207; P = 0.008), and SUA (β = 0.048 µmol/L, 95% CI = 0.001–0.026; P = 0.037) per 1 mmHg increase in DBP. Table 4 depicts the relationship between plasma markers and diastolic and systolic blood pressure.

**Table 4:** Association between the diastolic and systolic blood pressure and plasma markers.

Biomarkers	Entire Population			Men			Women		
	Β	95% CI(Lower Bound- Upper Bound)	P	β	95% CI(Lower Bound- Upper Bound)	P	β	95% CI(Lower Bound- Upper Bound)	P
**DBP**									
hs-CRP (mg/L)	−0.010	−0.108–0.061	0.587	−0.035	−0.172–0.040	0.225	0.013	−0.102–0.176	0.600
HCY (µmol/L)	0.073	0.080–0.258	0.000	0.092	0.064–0.272	0.002	0.050	0.002–0.343	0.047
NT-proBNP (ng/mL)	0.023	−0.001–0.004	0.229	0.013	−0.002–0.003	0.652	0.077	0.006–0.027	0.002
25-OHD (nmol/L)	0.039	0.002–0.124	0.044	0.048	−0.014–0.154	0.104	0.026	−0.042–0.137	0.299
HBA1c (%)	0.046	0.032–0.963	0.036	0.025	−0.460–0.942	0.500	0.062	0.070–1.325	0.029
hs-TnT (ng/L)	0.034	−0.008–0.090	0.100	0.053	−0.006–0.098	0.086	−0.004	−0.154–0.131	0.873
SUA (µmol/L)	0.037	−0.001–0.013	0.101	0.043	−0.003–0.017	0.182	0.022	−0.006–0.014	0.426
**SBP**									
hs-CRP (mg/L)	−0.029	−0.272–031	0.119	−0.037	−0.304–0.067	0.210	−0.029	−0.405–0.101	0.239
HCY (µmol/L)	0.076	0.156–0.475	0.000	0.113	0.174–0.536	0.000	0.030	−0.123–0.499	0.237
NT-proBNP (ng/mL)	0.019	−0.002–0.007	0.323	−0.014	−0.005–0.003	0.648	0.123	0.030–0.069	0.000
25-OHD (nmol/L)	0.002	−0.104–0.116	0.911	−0.001	−0.149–0.146	0.980	−0.007	−0.187–0.139	0.775
HBA1c (%)	0.098	1.017–2.687	0.000	0.083	0.193–2.63	0.023	0.108	1.092–3.378	0.000
hs-TnT (ng/L)	0.055	0.031–0.207	0.008	0.069	0.013–0.196	0.025	0.041	−0.064–0.454	0.141
SUA (µmol/L)	0.048	0.001–0.026	0.037	0.025	−0.010–0.024	0.431	0.047	−0.002–0.035	0.083

Adjusted for age, gender, cigarette status, Alcohol use, physical activity, BMI, high-density lipoprotein, LDL-C, triglycerides, creatinine, and Diabetes Mellitus.

Note: The plasma markers Beta estimates represents 1 unit the plasma biomarker measured in.

### The relationship between plasma markers and hypertension in the general population

After we compared the concentration of plasma markers between the control and hypertension group, we further calculated the adjusted OR to investigate the relationship between the plasma markers and hypertension. When the values of plasma markers were treated as continuous data, the adjusted OR and 95% CI for hypertension in the entire population for hcy and HBA1C were 1.02(1.01–1.04; P = 0.010) and 1.24(1.12–1.36; P < 0.001), respectively. Also, statistically significant associations between hcy and HBA1c concentrations and prevalence of hypertension were discovered in men and women. In addition, there was a statistically significant relationship between NT-proBNP levels and presence of hypertension in women (OR = 1.04, 95% CI: 1.02–1.06, P = 0.001). The association between the plasma markers and hypertension in the adjusted regression analyses based on the continuous variables is shown in Table 5. A similar result was observed when plasma markers included in regression models as quartiles. There was a positive association between the higher quartiles of CRP and the presence of hypertension after adjusting for lifestyle-related variables. However, this association was not maintained after adjustment for other explanatory variables such as lipid profile, BMI, and DM. The result of the present study showed that there exist positive associations between the higher quartiles of Hcy, NT-proBNP, and HBA1c concentrations and the presence of hypertension. (Table 6).

**Table 5:** ORs (95% CIs) of Hypertension when plasma markers used as continuous variables.

Biomarkers	Entire population			Men			Women		
	OR	95% CI	P	OR	95% CI	P	OR	95% CI	P
hs-CRP (mg/L)	0.997	0.98–1.01	0.681	1.00	0.98–1.02	0.985	0.99	0.97–1.02	0.460
HCY (µmol/L)	1.02	1.01–1.04	0.010	1.03	1.01–1.05	0.008	1.01	1.00–1.04	0.038
NT-proBNP (ng/mL)	1.00	1.00–1.00	0.669	1.00	1.00–1.00	0.963	1.04	1.02–1.06	0.001
25-OHD (nmol/L)	1.00	0.99–1.01	0.887	1.00	0.99–1.02	0.783	1.00	0.98–1.01	0.545
HBA1C (%)	1.24	1.12–1.36	<0.001	1.23	1.07–1.43	0.005	1.25	1.10–1.43	0.001
hs-TnT (ng/L)	1.00	0.99–1.01	0.984	1.00	0.99–1.01	0.745	0.99	0.97–1.02	0.438
SUA (µmol/L)	1.00	1.00–1.00	0.350	1.00	1.00–1.00	0.900	1.00	1.00–1.00	0.380

OR indicates odds ratio; CI, Confidence Interval; hs-CRP, high-sensitivity C-reactive protein; HbAC1, Glycosylated hemoglobin; NT-proBNP, N-terminal prohormone of brain natriuretic peptide; 25OHD, 25-Hydroxyvitamin D; hs-TnT, high-sensitivity Troponin T.

Adjusted for Age and gender, cigarette status, Alcohol use, physical activity, and BMI, total serum cholesterol, high-density lipoprotein, LDL-C, triglycerides, creatinine, and Diabetes Mellitus.

**Table 6:** Risk of Hypertension in the entire participants according to plasma marker quartiles.

Plasma Marker	Plasma markers			
	Quartile 1	Quartile 2	Quartile 3	Quartile 4
**hs-CRP**	**≤0.50**	**0.51–1.01**	**1.02–1.94**	**≥1.95**
Model 1	1.000(ref.)	1.35(1.08–1.67)^‡^	1.94(1.54–2.43)^‡^	1.83(1.46–2.30)^‡^
Model 2	1.000(ref.)	1.17(0.93–1.47)	1.51(1.19–1.91)^†^	1.30(1.02–1.66)*
Model 3	1.000(ref.)	1.10(0.88–1.39)	1.41(1.11–1.80)*	1.17(0.91–1.51)
**Hcy**	**≤7.97**	**7.98–9.47**	**9.48–11.92**	**≥1.93**
Model 1	1.000(ref.)	1.09(0.87–1.35)	1.22(0.97–1.53)	1.59(1.26–2.01)^‡^
Model 2	1.000(ref.)	1.06(0.84–1.33)	1.17(0.92–1.47)	1.52(1.19–1.94)^‡^
Model 3	1.000(ref.)	1.09(0.87–1.37)	1.17(0.92–1.47)	1.48(1.16–1.90)^†^
**hs-TnT**	**≤5.82**	**5.83–7.92**	**7.93–10.23**	**≥10.24**
Model 1	1.000(ref.)	1.15(0.91–1.46)	1.19(0.93–1.51)	1.21(0.94–1.55)
Model 2	1.000(ref.)	1.16(0.91–1.48)	1.16(0.91–1.49)	1.08(0.83–1.39)
Model 3	1.000(ref.)	1.16(0.91–1.49)	1.15(0.90–1.48)	1.05(0.81–1.37)
**NT-proBNP**	**≤30.75**	**30.75–50.77**	**50.78–83.79**	**≥83.80**
Model 1	1.000(ref.)	1.13(0.90–1.41)	1.06(0.85–1.32)	1.31(1.04–1.65)*
Model 2	1.000(ref.)	1.25(0.99–1.57)	1.18(0.93–1.49)	1.53(1.21–1.95)*
Model 3	1.000(ref.)	1.28(1.02–1.62)	1.24(0.98–1.56)	1.62(1.27–2.07)^‡^
**25-OHD**	**≤9.85**	**9.86–13.95**	**13.96–13.39**	**≥19.40**
Model 1	1.000(ref.)	0.81(0.65–1.01)	0.85(0.68–1.07)	0.99(0.79–1.25)
Model 2	1.000(ref.)	0.80(0.64–1.01)	0.84(0.66–1.06)	0.95(0.75–1.21)
Model 3	1.000(ref.)	0.81(0.65–1.03)	0.85(0.67–1.08)	0.97(0.76–1.24)
**HBA1C (%)**	**≤4.72**	**4.73–5.09**	**5.10–5.66**	**≥5.67**
Model 1	1.000(ref.)	1.08(0.87–1.34)	1.21(0.97–1.51)	2.31(1.84–2.90)^‡^
Model 2	1.000(ref.)	1.08(0.87–1.35)	1.21(0.97–1.50)	2.31(1.84–2.91)^‡^
Model 3	1.000(ref.)	1.06(0.85–1.33)^‡^	1.11(0.88–1.39)	1.94(1.49–2.52)^‡^
Model 4	1.000(ref.)	1.06(0.85–1.34)	1.10(0.88–1.39)	1.96(1.51–2.56)^‡^
**SUA** ^**#**^				
Model 1	1.000(ref.)	0.99(0.80–1.23)	1.08(0.87–1.35)	1.71(1.37–2.14)
Model 2	1.000(ref.)	0.90(0.72–1.12)	0.87(0.69–1.09)	1.23(0.97–1.56)
Model 3	1.000(ref.)	0.87(0.69–1.09)	0.83(0.66–1.05)	1.11(0.87–1.41)

*P < 0.05; ^†^P < 0.01; ^‡^P < 0.001.

^#^The cutoff points for the Serum Uric Acid were calculated for men and women separately, and the values are described in Tables 4 and 5.

hs-CRP = high-sensitivity C-reactive protein; hs-cTnT, high-sensitivity cardiac troponin T; NT-proBNP, N-terminal prohormone of brain natriuretic peptide; 25OHD, 25-Hydroxyvitamin D; SUA, serum uric acid and HbA1c, glycosylated hemoglobin A1c.

Model 1: Adjusted for age and gender.

Model 2: Adjusted for age, gender, smoking, Alcohol use, physical activity.

Model 3: Adjusted for the above plus total serum cholesterol, high-density lipoprotein, LDL-C, triglycerides and creatinine, BMI and Diabetes Mellitus.

Model 4: Sensitivity analysis for HBA1c markers.

### The relationship between plasma markers and hypertension in Men

We observed a significant relationship between serum Hcy and HbA1c levels and presence of hypertension after adjusting for multiple confounding factors, including age, cigarette status, alcohol use, physical activity, serum TC, HDL, LDL, TG, and creatinine (Model 2, in Table 7). The association between Hcy and HBA1c levels and presence of hypertension persisted even after BMI and diabetes mellitus were added to the model (Model 3 in Table 7).

**Table 7:** Risk of Hypertension in men according to plasma marker quartiles.

Plasma Marker	Plasma markers			
	Quartile 1	Quartile 2	Quartile 3	Quartile 4
**hs-CRP**	**≤0.55**	**0.56–1.04**	**1.05–2.07**	**≥2.08**
Model 1	1.000(ref.)	1.23(0.87–1.73)	1.66(1.17–2.36)^†^	1.70(1.20–2.42)^†^
Model 2	1.000(ref.)	1.12(0.79–1.59)	1.44(1.00–2.07)	1.41(0.98–2.04)
Model 3	1.000(ref.)	1.05(0.74–1.51)	1.36(0.94–1.96)	1.30(0.90–1.90)
**Hcy**	**≤8.94**	**8.95–10.92**	**10.93–13.48**	**≥13.49**
Model 1	1.000(ref.)	1.11(0.79–1.56)	1.36(0.96–1.92)	2.03(1.42–2.90)^‡^
Model 2	1.000(ref.)	1.07(0.75–1.52)	1.37(0.96–1.96)	2.03(1.40–2.94)^‡^
Model 3	1.000(ref.)	1.02(0.71–1.45)	1.36(0.95–1.96)	2.00(1.37–2.92)^‡^
**hs-TnT**	**≤6.48**	**6.49–8.95**	**8.96–11.72**	**≥11.73**
Model 1	1.000(ref.)	1.00(0.70–1.43)	1.16(0.80–1.66)	1.33(0.91–1.94)
Model 2	1.000(ref.)	1.06(0.74–1.53)	1.17(0.80–1.70)	1.36(0.92–2.00)
Model 3	1.000(ref.)	1.05(0.73–1.53)	1.09(0.75–1.60)	1.25(0.84–1.86)
**NT-proBNP**	**≤26.08**	**26.09–43.50**	**43.51–76.49**	**≥76.50**
Model 1	1.000(ref.)	1.08(0.76–1.53)	1.08(0.76–1.54)	1.15(0.80–1.67)
Model 2	1.000(ref.)	1.10(0.77–1.57)	1.15(0.80–1.65)	1.33(0.91–1.95)
Model 3	1.000(ref.)	1.15(0.80–1.66)	1.23(0.85–1.77)	1.40(0.95–2.07)
**25-OHD**	**≤12.04**	**12.05–16.50**	**16.51–22.69**	**≥22.70**
Model 1	1.000(ref.)	1.07(0.75–1.51)	1.33(0.94–1.90)	1.01(0.72–1.43)
Model 2	1.000(ref.)	1.10(0.77–1.57)	1.42(0.99–2.05)	1.09(0.76–1.56)
Model 3	1.000(ref.)	1.09(0.76–1.56)	1.40(0.97–2.02)	1.07(0.74–1.54)
**HbA1c (%)**	**≤4.71**	**4.72–5.10**	**5.11–5.70**	**≥5.71**
Model 1	1.000(ref.)	1.14(0.81–1.60)	1.16(0.82–1.64)	1.96(1.37–2.81)^†^
Model 2	1.000(ref.)	1.19(0.84–1.69)	1.17(0.82–1.67)	1.81(1.25–2.63)^†^
Model 3	1.000(ref.)	1.09(0.76–1.56)	1.07(0.74–1.53)	1.77(1.15–2.73)^†^
Model 4	1.000(ref.)	1.10(0.77–1.57)	1.01(0.70–1.46)	1.81(1.18–2.77)^†^
**SUA**	**≤303.36**	**303.37–353.11**	**353.12–403.82**	**≥403.83**
Model 1	1.000(ref.)	0.99(0.70–1.39)	1.11(0.79–1.58)	1.37(0.96–1.95)
Model 2	1.000(ref.)	0.92(0.65–1.31)	0.99(0.69–1.43)	1.12(0.77–1.64)
Model 3	1.000(ref.)	0.86(0.60–1.24)	0.93(0.64–1.34)	0.98(0.67–1.45)

*P < 0.05; ^†^P < 0.01;^‡^P < 0.001.

hs-CRP, high-sensitivity C-reactive protein; Hcy, Homocysteine; hs-cTnT, high-sensitivity cardiac troponin T; NT-proBNP, N-terminal prohormone of brain natriuretic peptide; 25OHD, 25-Hydroxyvitamin D; SUA, Serum uric acid; and HbA1c glycosylated hemoglobin A1c.

Model 1: Adjusted for age, cigarette status, Alcohol use, physical activity.

Model 2: Adjusted for the above plus total serum cholesterol, high-density lipoprotein, LDL-C, triglycerides, and creatinine

Model 3: Adjusted for the above plus BMI and Diabetes Mellitus.

Model 4: Sensitivity analysis for HBA1c.

### The relationship between plasma markers and hypertension in Women

We observed a positive and significant relationship between HbA1c, hs-CRP, Hcy, and NT-proBNP levels, and presence of hypertension after multivariate adjustment for potential confounders, including age, gender, cigarette status, alcohol use, physical activity, LDL, TC, HDL, TG, and creatinine (Model 1 and 2, in Table 8). The relationship between Hcy, NT-proBNP, and HbA1c concentrations and hypertension persisted even after adjusting for BMI and diabetes mellitus (Model 3 in Table 8). The fourth quartile of NT-proBNP concentrations was significantly associated with hypertension, with OR (95% CI) of 1.43(1.07–1.92), 1.63(1.20–2.21), and 1.85(1.35–2.53), for models 1, 2 and 3, respectively. In the fully adjusted multivariate analysis, the OR and 95% CI of hypertension for the participants in Q2, Q3, and Q4 compared to participants in the first quartile of HbA1c were 1.32(0.99–1.74), 1.79(1.33–2.41), and 2.37(1.70–3.30), respectively.

**Table 8:** Risk of Hypertension in women according to plasma marker quartiles in adjusted models.

Plasma Marker	Plasma markers			
	Quartile 1	Quartile 2	Quartile 3	Quartile 4
**hs-CRP**	**≤0.53**	**0.54–1.00**	**1.01–1.85**	**≥1.86**
Model 1	1.000(ref.)	1.31(0.99–1.75)	1.82(1.36–2.43)^‡^	1.94(1.45–2.59)^‡^
Model 2	1.000(ref.)	1.15(0.86–1.54)	1.60(1.19–2.16)^†^	1.53(1.13–2.08)^†^
Model 3	1.000(ref.)	1.02(0.75–1.38)	1.26(0.93–1.72)	1.04(0.75–1.45)
**Hcy**	**≤7.55**	**7.76–8.70**	**8.71–10.45**	**≥10.46**
Model 1	1.000(ref.)	1.21(0.91–1.61)	1.21(0.91–1.61)	1.53(1.15–2.05)^†^
Model 2	1.000(ref.)	1.23(0.92–1.64)	1.23(0.92–1.65)	1.53(1.13–2.07)^†^
Model 3	1.000(ref.)	1.18(0.87–1.59)	1.19(0.88–1.62)	1.39(1.02–1.90)*
**hs-TnT**	**≤5.45**	**5.46–7.26**	**7.27–9.42**	**≥9.43**
Model 1	1.000(ref.)	1.26(0.91–1.74)	1.05(0.76–1.45)	1.27(0.92–1.77)
Model 2	1.000(ref.)	1.29(0.92–1.79)	1.04(0.75–1.45)	1.27(0.91–1.78)
Model 3	1.000(ref.)	1.34(0.96–1.88)	1.02(0.73–1.43)	1.18(0.84–1.68)
**NT-proBNP**	**≤35.66**	**35.67–56.22**	**56.23–87.06**	**≥87.07**
Model 1	1.000(ref.)	1.11(0.83–1.48)	0.96(0.72–1.27)	1.43(1.07–1.92)*
Model 2	1.000(ref.)	1.22(0.90–1.63)	1.07(0.80–1.44)	1.63(1.20–2.21)^†^
Model 3	1.000(ref.)	1.29(0.95–1.75)	1.14(0.84–1.54)	1.85(1.35–2.53)^‡^
**25-OHD**	**≤8.60**	**8.61–12.30**	**12.21–16.83**	**≥16.84**
Model 1	1.000(ref.)	0.85(0.64–1.14)	0.77(0.58–1.03)	0.96(0.72–1.28)
Model 2	1.000(ref.)	0.87(0.65–1.17)	0.80(0.59–1.07)	0.95(0.70–1.28)
Model 3	1.000(ref.)	0.84(0.62–1.14)	0.76(0.56–1.03)	0.86(0.63–1.17)
**HBA1C (%)**	**≤4.72**	**4.73–5.09**	**5.10–5.11**	**≥5.12**
Model 1	1.000(ref.)	2.45(1.79–3.34)	1.36(1.02–1.81)*	1.01(0.76–1.35)^‡^
Model 2	1.000(ref.)	1.06(0.80–1.42)	1.34(1.00–1.78)*	2.21(1.61–3.05)^‡^
Model 3	1.000(ref.)	1.32(0.99–1.74)	1.79(1.33–2.41)^‡^	2.37(1.70–3.30)^‡^
Model 4	1.000(ref.)	1.32(1.00–1.75)	1.83(1.36–2.48)^‡^	2.35(1.63–3.39)^‡^
**Uric Acid**	**≤243.55**	**243.56–281.03**	**281.04–328.26**	**≥328.27**
Model 1	1.000(ref.)	0.96(0.72–1.28)	0.99(0.75–1.32)	1.89(1.41–2.53)^‡^
Model 2	1.000(ref.)	0.89(0.66–1.19)	0.88(0.65–1.18)	1.56(1.13–2.13)^†^
Model 3	1.000(ref.)	0.87(0.64–1.17)	0.76(0.56–1.04)	1.20(0.87–1.67)

*P < 0.05; ^†^P < 0.01;^‡^P < 0.001.

hs-CRP, high-sensitivity C-reactive protein; Hcy, Homocysteine; hs-cTnT, high-sensitivity cardiac troponin T; NT-proBNP, N-terminal prohormone of brain natriuretic peptide; 25OHD, 25-Hydroxyvitamin D; SUA, Serum uric acid; and HbA1c glycosylated hemoglobin A1c.

Model 1: Adjusted for age, cigarette status, Alcohol use, physical activity.

Model 2: Adjusted for the above plus total serum cholesterol, high-density lipoprotein, LDL-C, triglycerides, and creatinine.

Model 3: Adjusted for the above plus BMI and Diabetes Mellitus.

Model 4: Sensitivity analysis for HBA1c.

### Sensitivity analysis

To investigate whether there was a possibility that the interaction effect between hypertension status and DM on plasma HBA1c levels might have influenced the predictive power of HBA1C values for hypertension, we calculated the multivariable-adjusted odds ratios and 95% CIs in non-diabetes individuals (N = 2444). Those individuals with a high HBA1c level had a greater likelihood of having hypertension. This relationship persisted even after full adjustment for potential confounders. The OR and 95% CI for the sensitivity analysis is included in Tables 6, 7 and 8 for the entire population, men, and women, respectively.

## Discussion

In this cross-sectional study, we report significant positive associations between plasma markers including, Hcy, NT-proBNP, and HbA1c, and the prevalence of hypertension in middle and old age population. These associations were independent of potential confounders; age, gender, BMI, diabetes, alcohol use, cigarette status, physical activity, TC, HDL, LDL, TG, and creatinine.

Our study reported that elevated Hcy confers a corresponding increase in DBP, SBP, and prevalence of hypertension. The results of the current study were robust to Hcy distribution and were similar when Hcy were included in regression models in quartiles. It has been previously demonstrated that hyperhomocysteinemia contributes to endothelial cell injury, increases the rigidity of vessels, and eventually influences the process of normal blood flow of haemostasis adversely^33^, indicating that elevated Hcy adds to inflammation and vascular remodeling during the initiation and progression of hypertension. In our study, the participants in the fourth quartile of Hcy had a significantly increased prevalence of hypertension, confirming that the risk of hypertension increases with an increase of Hcy. This finding highlights the potential role of total Hcy in the risk stratification of hypertension.

In the past, HbA1c has not been considered as a risk marker for hypertension. However, HbA1c is the most significant regulatory marker established for diabetic care. In the present study, we observed a significant and positive relationship between HbA1c values and hypertension, even after multivariate adjustment for conventional cardiovascular risk factors. According to a previous cohort study, HbA1c values are positively linked to systolic BP dipping percent among adults who were both obese and pre-diabetic but were absent in obese adults who were free of pre-diabetic^34^, highlighting that HbA1c is closely linked with BMI. In the present study, BMI was significantly higher in the hypertension group compared with control. However, our study controls the differences in total cholesterol level between hypertension and control groups. Thus, the strong association of HbA1c with hypertension may not be limited to cholesterol levels. The multivariable odds ratios of hypertension across the 2^nd^ to 4^th^ compared to the 1^st^ quartiles of HBA1c were continuously surged in models 1, 2 and 3. This association marginally diminished, but still maintained its significance in the highest quartiles of serum HBA1c when DM and BMI were added into the regression models. This suggests that the strong inter-relationships between these two independent variables carry significant weight on the regression model results. Furthermore, there was a significant interaction between hypertension status and DM on HBA1c levels in our study. However, this interaction did not significantly influence the predictive power of HBA1c values for hypertension. This is corroborated in the multivariable-adjusted model in non-diabetes individuals showing a positive association between elevated HBA1c levels and hypertension. Bearing in mind that HBA1c is closely linked with multiple features of metabolic syndrome which relate with insulin resistance including, obesity and hyperlipidemia, pre-diabetes, and DM, a follow-up study is essential to confirm the positive, independent and robust association of elevated HBA1c with the presence of hypertension.

It has been previously reported that there exists a positive association between C-reactive protein and hypertension risk in women^35^. However, we observed no significant association between hs-CRP marker and hypertension, an observation consistent with a previous study^25^. Likewise, plasma hs-cTnT has been suggested to complement other diagnostic biomarkers in predicting prehypertension. Also, an elevated hs-cTnT has been indicated as a predictor of cardiovascular outcome in hypertensive individuals and the general population, which made it an important biomarker in patients with acute cardiac ischemia^36^. A recent study reported that hs-cTnT level positively correlates with prehypertension and underscored hs-cTnT as an independent predictor of prehypertension^17^. In our study, the hs-cTnT values were markedly higher in hypertension compared with the control group. However, we found no significant relationship between hs-cTnT levels and hypertension, particularly after adjustment for potential confounding variables.

NT-proBNP concentrations have been suggested to connect with diastolic dysfunction and left ventricular hypertrophy in hypertensive individuals^37^, extending the clinical application of this biomarker beyond that of heart failure. The randomized TEAMSTA Protect I- Trial reported that NT-proBNP concentrations were significantly reduced 6 months after therapy initiation from 64.8 to 53.3 ng/L^38^, suggesting its association with cardiovascular risk reduction. The present results showed obviously elevated NT-proBNP concentrations in the hypertension group compared with controls. A previous report from Atherosclerosis Risk in Communities Study (ARICS) concluded that persons with raised NT-proBNP were at greater risk of hypertension^39^. Our study demonstrated a positive association between NT-proBNP concentrations and SBP and DBP values, and hypertension prevalence, particularly in the general population and in women but not in men. In addition, a survey from the Uranosaki cohort study reported a positive association of NT-proBNP concentration with SBP among females^40^. This finding suggests that the outcome of our data in the general population was influenced by gender. Therefore, gender-specific evaluation is strictly required during NT-proBNP analysis. The findings, which are particular for women, highlight that future studies are needed to explore the underlying biological mechanisms of the observed association.

A previous study reported that depleted serum 25-OHD was negatively linked with hypertension among Finnish men^41^. Also, a recent study concluded that lower 25OHD was associated with higher blood pressure especially in those who use blood pressure medications^42^. However, our data reported no significant relationship between 25OHD values and presence of hypertension.

The present study is the first observational survey that determined the level of a number of CVD markers simultaneously and explored the relationship between these markers and hypertension in the middle-aged and elderly Chinese population in Dalian, Northeast China. However, several limitations can be stated for this study. The main drawback of our study is its cross-sectional nature due to which we are unable to ascribe causality to the observed relationship. The HbA1c values were astonishingly low and our study did not have control over the potential modification effect of small dense of low-density lipoprotein, Apo-lipoproteins, and BMI, which may deviate the true predictive power of HBA1c. However, we considered the interaction analysis between hypertension and diabetes status on HBA1c levels. Also, the use of echocardiographic data would have strengthened the validity of our associations; for instance, differences in left ventricular ejection fraction (LVEF) could be of importance when looking at the concentrations of NT-proBNP, hs-TnT, and HBA1c.

In conclusion, we found strong independent associations between homocysteine and HBA1c concentrations and prevalence of hypertension and high BP levels in the middle-aged and elderly Chinese population. The gender-specific data replicated positive associations between homocysteine, NT-ProBNP, and HBA1c concentrations and prevalence of hypertension in women, even after full adjustment for the potential confounding variables. Also, the relationship between homocysteine and HBA1c levels and the presence of hypertension in men was robust to potential confounding variables. For the use of these plasma markers in the prevention and management of high BP, future studies to explore the underlying biological mechanisms of the observed association, and longitudinal studies to validate the relationship are needed.
